# Investigating dynamics in attentive and inattentive responding together with their contextual correlates using a novel mixture IRT model for intensive longitudinal data

**DOI:** 10.1111/bmsp.12373

**Published:** 2024-12-07

**Authors:** Leonie V. D. E. Vogelsmeier, Irina Uglanova, Manuel T. Rein, Esther Ulitzsch

**Affiliations:** ^1^ Tilburg University Tilburg The Netherlands; ^2^ IPN—Leibniz Institute for Science and Mathematics Education Kiel Germany; ^3^ Centre for Educational Measurement University of Oslo Oslo Norway; ^4^ Centre for Research on Equality in Education University of Oslo Oslo Norway

**Keywords:** careless responding, ecological momentary assessment, intensive longitudinal data, latent Markov modelling, mixture modelling, three‐step approach, time series

## Abstract

In ecological momentary assessment (EMA), respondents answer brief questionnaires about their current behaviours or experiences several times per day across multiple days. The frequent measurement enables a thorough grasp of the dynamics inherent in psychological constructs, but it also increases respondent burden. To lower this burden, respondents may engage in careless and insufficient effort responding (C/IER), leaving data contaminated with responses that do not reflect what researchers want to measure. We introduce a novel approach to investigating C/IER in EMA data. Our approach combines a confirmatory mixture item response theory model separating C/IER from attentive behaviour with latent Markov factor analysis. This enables gauging the occurrence of C/IER and studying transitions among states of different response behaviours including their contextual correlates. The approach can be implemented using R packages. An empirical application showcases the approach's efficacy in pinpointing C/IER instances and gaining insights into their underlying causes. We showcase that the approach identifies various C/IER response patterns but requires heterogeneous and negatively worded items to detect straightlining. In a simulation investigating robustness against unaccounted for changes in measurement models underlying attentive responses, the approach proved robust against heterogeneity in loading patterns but not against heterogeneity in factor structures. Extensions to accommodate the latter are discussed.

## 1 INTRODUCTION

Intensive longitudinal data collected via methods like ecological momentary assessment (EMA) have great potential for studying the dynamics of psychological constructs such as well‐being in daily life (Scollon et al., 2009). In EMA, respondents answer brief questionnaires about their current behaviours or experiences in daily‐life situations several times per day over a prolonged period of time via mobile phone apps. The frequent and repetitive measures provide researchers and practitioners with valuable insights, enabling a thorough grasp of the dynamics inherent in psychological traits or constructs (Myin‐Germeys & Kuppens, 2021; Wrzus & Mehl, 2015).

EMA's demanding sampling schedule, however, also increases respondents' burden and, as a consequence, may decrease their willingness and/or ability to put effort into completing the study, rendering EMAs vulnerable to respondent non‐compliance (Dejonckheere & Erbas, 2021). In the present study, we focus on careless and insufficient effort responding (C/IER)^1^ as a prominent form of respondent non‐compliance. C/IER occurs when respondents complete questionnaires without making the effort to carefully evaluate the administered items (Huang et al., 2015; Ulitzsch et al., 2024). As a consequence, EMA data may be contaminated with responses that do not reflect what researchers want to measure.

Having techniques that allow one to identify and investigate (dynamics in) C/IER in EMA data is important for at least two reasons. Firstly, from a methodological perspective, detecting C/IER is vital for accurate inferences about the dynamics of psychological constructs (DeSimone et al., 2018; Huang et al., 2012; Kam & Meyer, 2015; Schmitt & Stuits, 1985; Woods, 2006). C/IER rates as low as 5% that remain undetected and unaccounted for can constitute psychometric properties of the questionnaires and introduce bias in the correlations between measured constructs (Huang et al., 2015; McGrath et al., 2010). Consequently, ignoring C/IER can result in inaccurate interpretations and hinder the replication of research findings (Curran, 2016).

Secondly, from a substantive point of view, C/IER identification makes it possible to study person and contextual characteristics related to its occurrence. This may increase researchers' awareness of contexts that, due to an increased risk of C/IER and the resulting lowered data quality, are hard to study and for which further incentives may be introduced to increase data quality. This is particularly relevant for EMA because individuals complete questionnaires in daily life and may be exposed to environmental distractions that may impede their capacity to respond attentively. For instance, if respondents cannot concentrate, perhaps because they are preparing for an important work presentation later that day, they may engage in C/IER.^2^ Further, understanding determinants of C/IER may ultimately aid in designing EMA studies that curb its occurrence and yield data of higher quality. For instance, researchers can investigate how sampling schedules affect C/IER occurrence and consider conclusions drawn from these investigations when designing EMAs.

Although studied excessively in cross‐sectional research (Bowling et al., 2016; Huang et al., 2015; Maniaci & Rogge, 2014; McKay et al., 2018; Nichols & Edlund, 2020), only a few studies have focused on C/IER in EMA (Eisele et al., 2022; Hasselhorn et al., 2022, 2023; Jaso et al., 2022), with one possible reason being that tools for identifying and investigating C/IER in EMA data are still scarce. Research aiming to detect C/IER in EMA data has predominantly relied on established behavioural indicators for C/IER detection (see Curran, 2016; Meade & Craig, 2012; Niessen et al., 2016, for overviews in the context of cross‐sectional data). These approaches are limited, however, in that they rely on somewhat arbitrary threshold settings separating attentive from C/IER behaviour and do not take C/IER identification uncertainty into account. While mixture modelling approaches overcoming these limitations constitute a rich body of research in the cross‐sectional context (e.g., Arias et al., 2020; Ulitzsch, Pohl, et al., 2022; Ulitzsch, Yildirim‐Erbasli, et al., 2022; van Laar & Braeken, 2022), model‐based approaches tailored to the EMA context emerged only recently and have, so far, only been developed for collateral information from screen time data (Ulitzsch et al., 2024).

In this study, we aim to expand EMA researchers' toolkit with a response‐pattern‐based mixture modelling approach for investigating dynamics in attentive and inattentive responding together with their contextual correlates. To this end, we blend *confirmatory* mixture item response theory (IRT) models developed for cross‐sectional research (Uglanova et al., in preparation) with latent Markov factor analysis (LMFA; Vogelsmeier, Vermunt, van Roekel, & De Roover, 2019), which was originally developed for EMA to support investigations of transition patterns between *exploratorily* identified changes in the measurement model (i.e., changes in the extent to which items measure constructs).

In what follows, we first review current approaches to C/IER detection in EMA data. Next, we provide a short introduction to LMFA and present an approach that substitutes LMFA's exploratory identification of measurement model changes with theory‐based IRT component models for attentive and inattentive behaviour. We delineate how the proposed approach can easily be implemented with standard R packages. Using real EMA data, we illustrate the method and showcase how it allows studying transition patterns between attentive and inattentive responding and their contextual correlates.

We evaluate the approach in three simulation studies. In the first study, we investigate the trustworthiness of C/IER detection under typical, but challenging, conditions that may impede the separability of attentive and inattentive item responses. We conclude that the method performs well even under challenging conditions but requires heterogeneous and negatively worded items to detect straightlining. Then, in two simulation studies, we evaluate robustness against violations of the key assumption that the same measurement model holds across all attentive observations. From these simulation studies we conclude that C/IER identification is robust against unaccounted heterogeneity in attentive loading patterns across observations but not against unaccounted for heterogeneity in the factor structure underlying attentive responses. We conclude by discussing extensions to accommodate changes in attentive measurement models.

## 2 C/IER DETECTION IN EMA DATA

### 2.1 Attention check and bogus items

Attention check and bogus items are items that elicit a specific attentive response (such as disagreement with “I enjoy eating cement” or compliance with the prompt “Choose response option 5”), such that deviations from this response can be assumed to signal C/IER. However, these items are unsuitable for fine‐grained monitoring of C/IER in EMAs, as repeated and extensive administration may confuse attentive respondents (Meade & Craig, 2012). In addition, attention check and bogus items can be suspected of having very low power. For instance, in an EMA survey with 163 respondents, Eisele et al. (2022) found only four respondents failing instructional manipulation check items more than once, while multiple other indicators signalled markedly higher C/IER proportions.

### 2.2 Indicators based on response patterns and screen times

Behavioural indicators based on response patterns of collateral information scan these for aberrations presumably signalling C/IER. Using the long‐string index (Johnson, 2005) to scan for suspiciously low response variability or the exclusion of observations associated with screen times that are too short to properly evaluate the administered items (see Hasselhorn et al., 2022, for an application to EMA data) are well‐known examples. Another example is person‐fit indices (for an overview, see Karabatsos, 2003). These indices assess the degree to which individuals' responses deviate from the most typical response behaviour in the sample or how they adhere to a predefined model in particular (e.g., an IRT or factor model, Mansolf & Reise, 2017; Roman et al., 2024) estimated using all data. When using such indicators, researchers must decide on thresholds separating attentive behaviour from C/IER. This decision is ultimately an arbitrary one, with even minor differences in threshold settings often heavily impacting conclusions on C/IER contamination (Niessen et al., 2016; Ulitzsch, Domingue, et al., 2023; Ulitzsch, Shin, & Lüdtke, 2023).

Data‐driven approaches to identifying C/IER observations with response‐pattern‐based and screen time indicators mitigate the subjectivity of threshold settings. Jaso et al. (2022) suggested setting thresholds such that the cleaned data set minimizes implausible positive correlations among psychometric antonyms (e.g., relaxed and anxious). Further, latent class analysis on one or more indicators can be employed (see Kam & Meyer, 2015; Maniaci & Rogge, 2014, for applications in the cross‐sectional context; and Hasselhorn et al., 2023, for a multilevel extension for EMA data), entirely avoiding the setting of thresholds. In this approach, classes with aberrant conditional means on the analysed indicators are labelled as C/IER. Note, however, that post hoc interpretation of the obtained latent classes may not always be straightforward (see Ulitzsch et al., 2024, for further examples and discussions). Hence, despite these recent advances, a common limitation of approaches relying on behavioural indicators remains that they are only loosely grounded in subject‐matter theory on respondent behaviour. This renders them susceptible to ambiguity and arbitrariness – either in setting thresholds or post hoc interpretation of latent classes.

### 2.3 Confirmatory mixture model based on screen times

As an alternative to post hoc interpretations of latent classes as attentive or careless, *confirmatory* mixture models can be employed. These translate theoretical considerations on respondent behaviour into two mixture component models – one representing an assumed attentive and the other an assumed inattentive data‐generating process. Posterior class probabilities obtained from the resultant two‐class mixture model can then be employed to draw conclusions on the attentiveness of a given observation.

Such models have been developed predominantly for cross‐sectional item response data (e.g., Arias et al., 2020; Kam & Cheung, 2023; Ulitzsch, Yildirim‐Erbasli, et al., 2022; van Laar & Braeken, 2022). In these models, attentive item responses are assumed to reflect the to‐be‐measured traits, i.e., to follow standard measurement models for item response data – also referred to as latent trait models – such as confirmatory factor analysis models (Arias et al., 2020; Kam & Cheung, 2023) or IRT models for polytomous data (Ulitzsch, Domingue, et al., 2023; Ulitzsch, Pohl, et al., 2022; van Laar & Braeken, 2022). Inattentive item responses, in contrast, are assumed to be unrelated to the items' content (as respondents do not invest effort into evaluating the items) and the traits to be measured (because respondents do not invest effort in selecting relevant response options). Instead, inattentive item responses are assumed to be driven by respondents' category preferences (Arias et al., 2020), to be purely random (van Laar & Braeken, 2022), or both (Kam & Cheung, 2023; Uglanova et al., in preparation).

Only recently, Ulitzsch et al. (2024) provided a confirmatory mixture modelling approach for C/IER tailored to the EMA context. This model is formulated for screen times instead of for item responses. Screen times can easily be recorded in electronically administered EMAs and indicate how much time respondents required to interact with the items presented on a given screen. Hence, the component models for this confirmatory mixture model reflect theoretical considerations on how this time may evolve in EMAs. For attentive screen times, the model assumes an exponential decay process, capturing possible speed‐ups due to familiarization and practice with the EMA delivery system and the administered measures. Inattentive screen times are assumed to randomly fluctuate and to be, on average, shorter than screen times of attentive respondents who familiarized themselves with the EMA. The model allows attentiveness to vary on the respondent‐by‐occasion level and can be enriched with person‐ and occasion‐level covariates, thereby providing a sophisticated tool to investigate time‐invariant (e.g., affinity to technology) and time‐varying (e.g., time of the day) covariates of C/IER occurrence.

Nevertheless, this model‐based approach is of somewhat limited practical applicability. Firstly, it comes with strong requirements for EMA data collection. For instance, it is not clear yet how to handle routing designs. These pose a challenge because they result in questionnaires that vary in length across respondents and, as such, in the time attentive respondents require. Secondly, due to its complexity, the approach is not very easy to apply, and estimation issues are likely to occur. Thirdly, having only recently been developed, validity evidence that this approach indeed accurately captures C/IER is still pending. In this study, we aim to complement this approach with an easily applicable approach leveraging item responses. To this end, we integrate confirmatory mixture IRT models for cross‐sectional data with LMFA originally developed for exploratory investigations of EMA measurement model changes.

### 2.4 Exploratory mixture model based on item responses for exploring measurement model changes

The recently proposed LMFA (Vogelsmeier, Vermunt, van Roekel, & De Roover, 2019) is an exploratory mixture model that classifies individual‐ and timepoint‐specific observations from EMA data into latent states based on differences in response behaviour. Different response behaviours are modelled with measurement models identified with either exploratory factor analysis for continuous data (Vogelsmeier, Vermunt, van Roekel, & De Roover, 2019) or exploratory IRT models for ordinal data (Vogelsmeier, Vermunt, Keijsers, & De Roover, 2021). Transitions between the latent states representing the different response behaviours are captured via a latent Markov model (LMM), which sheds light on the overall probabilities of transitioning to a particular state. These probabilities can be related to covariates capturing individual or situational characteristics. LMFA thus holds promise in unveiling the moments when individuals transition between different response behaviours and identifying correlates with individual and situational characteristics. Its major advantages lies in its flexibility, as it is capable of uncovering manifold changes in measurement models. Vogelsmeier, Cloos, et al. (2023), for instance, identified two affect structures and identified contextual (negative event intensity) and person characteristics (neuroticism) related to transition patterns between affect structures. In principle, LMFA could also be applied to uncover C/IER: Exploring what the state‐specific measurement models and the relationships between the covariates and state memberships look like may reveal that one state corresponds to attentive responding and another to C/IER. However, as highlighted by Vogelsmeier (2022). LMFA is currently not tailored to distinguish between attentive responding and C/IER as the method merely differentiates between any apparent difference in response behaviour, and post hoc interpretation of the latent states can be ambiguous.

### 2.5 Proposed method: LMFA with confirmatory mixture IRT models

In this article, we combine LMFA (originally developed to identify and explore measurement model changes and their contextual correlates in EMA data) with theory‐based confirmatory mixture IRT models for C/IER (originally developed for detecting C/IER in cross‐sectional data). The new methodology optimally leverages item information by clustering observations into two states that are in advance defined as attentive responding and C/IER using specific measurement model constraints. This overcomes the need for post hoc interpretations of possibly ambiguous latent states resulting from the exploratory models used in regular LMFA. Like previously developed mixture modelling approaches to C/IER in EMA data (Hasselhorn et al., 2023; Ulitzsch et al., 2024), this new method does not require decisions on threshold settings and takes C/IER classification uncertainty into account. The method is applicable to EMA studies employing multiple items with ordinal response scales to measure psychological constructs. Possible extensions are discussed in the Discussion section.

## 3 METHOD

The proposed methodology builds upon the traditional LMFA model that comprises two parts: (1) the state‐specific measurement models determining the nature of the relations between items and constructs, which are obtained via exploratory mixture factor analysis models (Vogelsmeier, Vermunt, van Roekel, & De Roover, 2019) or exploratory ordinal IRT models (Vogelsmeier, Vermunt, Keijsers, & De Roover, 2021) and (2) the latent Markov transition model describing how individuals transition between the latent measurement‐model states over time and how these transitions correlate with individual‐ or context‐specific characteristics. What distinguishes our proposed methodology from the regular LMFA is the measurement model specification, which is no longer exploratory but confirmatory. Specifically, the presence of relationships between items and constructs are determined in advance using theory‐based confirmatory mixture IRT modelling specifically tailored to capture attentive responding versus C/IER. In the following, we first explain the data structure, then detail the measurement model specification, and finally explain the transition model specification.

### 3.1 Data structure

We assume intensive longitudinal data with observations nested within individuals. For every measurement occasion, we consider scales with G+1 ordered response categories. We denote by $y_{ijt}\in \{0,\text{…},G\}$ the response of respondent i∈{1,…,N} to item j∈{1,…,J} on measurement occasion t∈{1,…,T}. The J items are assumed to measure a set of M substantive traits. Note that the number of timepoints T typically differs across respondents. However, we omit index i in $T_{i}$ for simplicity. The observations are collected in the 1×J vectors $y_{it}=(y_{i1t},\text{…},y_{iJt})$, which are collected in the T×J subject‐specific data matrices $Y_{i}={\left(y_{i1}^{\prime },\text{…},y_{iT}^{\prime }\right)}^{\prime }$. The data matrices are concatenated in the dataset $Y={\left(Y_{1}^{\prime },\text{…},Y_{N}^{\prime }\right)}^{\prime }$, with $\sum_{i=1}^{N}T_{i}$ rows.

### 3.2 Measurement model

To disentangle inattentive from attentive responding, we employ the two‐state confirmatory mixture IRT model proposed by Uglanova et al. (in preparation), which incorporates previous IRT models for C/IER (Ulitzsch, Pohl, et al., 2023; Ulitzsch, Yildirim‐Erbasli, et al., 2022; van Laar & Braeken, 2022) as special cases. We assume that at each measurement occasion t, respondent i can be in one of two latent states k∈{1,2}. The state memberships are indicated via the binary indicators $s_{itk}$. These are equal to 1 for state k and equal to zero for the other state. Specifically, $s_{it1}=1$ denotes an attentive state membership and $s_{it2}=1$ denotes an inattentive state membership.

Attentive responses are assumed to follow a (possibly multidimensional) graded response model (GRM; Samejima, 1969, 2016). That is, the probability that an attentive respondent i will select response option g or higher on item j on measurement occasion t is modelled as

(1) $$\begin{array}{ll} p(y_{ijt}\geq g|s_{it1}=1)=\frac{\exp(\sum_{m}^{M}\alpha_{jm}\theta_{imt}+\kappa_{jg})}{1+\exp(\sum_{m}^{M}\alpha_{jm}\theta_{imt}+\kappa_{jg})} & \;\text{for}\;g\in \{1,\text{…},G\},\\ p(y_{ijt}\geq g|s_{it1}=1)=1 & \;\text{for}\;g=0,\\ p(y_{ijt}\geq g|s_{it1}=1)=0 & \;\text{for}\;g=G+1,\; \end{array}$$

where $\alpha_{jm}$ gives the loading parameter of item j on the substantive trait m, $\theta_{imt}$ gives respondent i's location on trait m at the tth measurement occasion, and $\kappa_{jg}$ is the gth category threshold for item j. Note that raw item responses are modelled, i.e., item responses to negatively worded items are not recoded. Hence, in the case where there are negatively worded items in the administered scales, loading parameters $\alpha_{jm}$ can be expected to take negative values. For model identification, latent trait expectations and variances are set to 0 and 1 respectively.

The probability that an attentive respondent i will select response option g on item j on measurement occasion t can simply be obtained as

(2) $$p(y_{ijt}=g|s_{it1}=1)=p(y_{ijt}\geq g|s_{it1}=1)-p(y_{ijt}\geq g+1|s_{it1}=1).$$

When inattentive ($s_{it2}=1$), respondents are assumed to provide responses that are not reflective of the to‐be‐measured substantive traits. Instead of being elicited by the items' content, inattentive responses are assumed to be driven by mere category preferences that generalize across all items administered at measurement occasion t. In the inattentive measurement model, this assumption is incorporated by substituting the possibly multidimensional substantive traits with a unidimensional trait that captures respondents' category preferences. Hence, a unidimensional GRM measuring respondent i's category preferences $\xi_{it}$ at measurement occasion t is assumed, where all loadings are set to 1 and thresholds are set to be the same across items, i.e.,

(3) $$\begin{array}{ll} p(y_{ijt}\geq g|s_{it2}=1)=\frac{\exp(1\xi_{it}+\kappa_{g})}{1+\exp(1\xi_{it}+\kappa_{g})} & \;\text{for}\;g\in \{1,\text{…},G\}.\; \end{array}$$

Note that, because inattentive respondents are assumed to not pay attention to items' content, loadings for both positively and negatively worded items are set to 1. Hence, analysing scales with both positively and negatively worded items facilitates the detection of C/IER because the attentive and inattentive component models will have markedly different loading structures, especially in conditions that otherwise challenge C/IER identification, for instance, when unidimensional scales are modelled. Unidimensional scales present unique challenges compared to multifactor scales because, in the latter, the presence of (particularly negative) correlations between factors helps distinguish careless from attentive responding, as behaviours like straightlining would produce a perfect positive relationship between factors.^3^ Note that the imposed constraints can be seen as the IRT equivalent of the factor mixture modelling approach proposed by Kam and Cheung (2023).

Based on theoretical considerations and simulation evidence, Uglanova et al. (in preparation) demonstrated that the inattentive component model could accommodate different types of careless behaviour – spanning both random responding as well as pronounced respondent‐specific category preferences. For instance, when all respondents exhibit random responding, var(ξ)≈0. In this case, with the latent trait expectation set to zero, Equation (3) essentially reduces to

(4) $$\begin{array}{ll} p(y_{ijt}\geq g|s_{it2}=1)=\frac{\exp(\kappa_{g})}{1+\exp(\kappa_{g})} & \;\text{for}\;g\in \{1,\text{…},G\},\; \end{array}$$

and thresholds $\kappa_{g}$ determine the category probabilities of random response behaviour.

**FIGURE 1:**
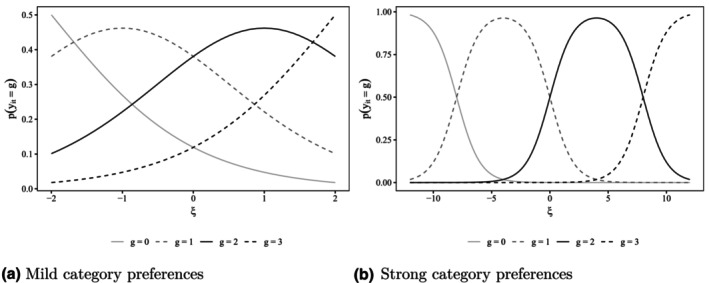
Illustrative item characteristic curves for different types of C/IER behaviour. ξ: category preference, (a) Mild category preferences, (b) Strong category preferences.

Figure 1 illustrates different scenarios of inattentive respondents differing in their category preferences. In Figure 1a, inattentive respondents exhibit mildly pronounced category preferences, i.e., depending on their location on the latent category preference trait, tend to slightly favour lower, middle, or upper response categories. Figure 1b, in contrast, depicts a scenario where inattentive respondents have a strong preference for a specific category, i.e., exhibit behaviour that closely resembles straightlining. Here, category boundaries are further spaced apart, and var(ξ) is larger than in the scenario depicted in Figure 1a.

Note that the aim of this component model is not to disentangle different types of C/IER but to “absorb” as many different C/IER response patterns as possible, provide a trustworthy estimate of the overall C/IER rate, and account for the presence of C/IER in the estimation of attentive parameters. Indeed, Uglanova et al. (in preparation) showed that even though their proposed component model is misspecified as soon as there are different types of C/IE patterns, it is perfectly capable of capturing a blend of different types of patterns, for instance, when some respondents exhibit random responding and others have strongly pronounced category preferences. Given that the C/IER component model is likely misspecified, we advise against interpreting the latent category preference variable. Rather, we recommend inspecting the C/IER component model parameters to get an impression of the marginal distribution of C/IER responses across all captured C/IER patterns.

### 3.3 Transition model

The transition model describes how individuals transition between the attentive and C/IER states and is obtained using LMM (e.g., Bartolucci et al., 2014), which is defined by three distinct types of parameters: the initial state, transition, and response probabilities. Together, the parameters form the joint distribution of the observations and states for subject i:

(5) $$\begin{array}{ll} & p(Y_{i},S_{i}|Z_{i})=p(y_{i1},\text{…},y_{iT},s_{i1},\text{…},s_{iT}|z_{i1},\text{…},z_{iT})\\ & =\overset{\text{initial state probabilities}}{\overbrace{p(s_{i1}|z_{i1})}}\overset{T}{\underset{t=2}{\prod }}\overset{\text{transition probabilities}}{\overbrace{p_{\kappa_{ti}}(s_{it}|s_{it-1},z_{it})}}\overset{T}{\underset{t=1}{\prod }}\overset{\text{response probabilities}}{\overbrace{p(y_{it}|s_{it})}}.\; \end{array}$$

Note that the 2×1 vectors $s_{it}={\left(s_{it1},s_{it2}\right)}^{\prime }$ contain the binary indicators $s_{itk}$. The U×1 vectors $z_{it}={\left(z_{it1},\text{…},z_{itU}\right)}^{\prime }$ comprise the covariate values $z_{itu}$, with u=1,…,U, referring to the subject‐ and timepoint‐specific covariates possibly influencing the initial or transition probabilities as described below. The state‐specific response probabilities $p(y_{it}|s_{itk}=1)$ indicate the probability for the attentive and C/IER response patterns at timepoint t given the state membership at that timepoint, $s_{i1k}=1$. These probabilities depend on the two state‐specific models as described earlier.

The initial state probabilities indicate the probabilities of starting in state k at timepoint t=1. The probabilities can depend on covariate values at the first timepoint, indicated as $z_{i1}$, and the probabilities are collected in a 2×1 probability vector π with elements $\pi_{k}=p(s_{i1k}=1|z_{i1})$ and $\sum_{k=1}^{2}\pi_{k}=1$. The initial state probabilities are typically modelled via a logit model to prevent parameter range restrictions:

(6) $$\log\left(\frac{p(s_{i1k}=1|z_{i1})}{p(s_{i11}=1|z_{i1})}\right)=\beta_{0k}+\beta_{k}^{\prime }z_{it=1},$$

where the initial state intercepts are denoted by $\beta_{0k}$ and the initial state slopes that quantify the effect of the covariates on the initial state memberships are captured by the vectors $\beta_{k}^{\prime }={\left(\beta_{k,Z_{i11}},\text{…},\beta_{k,Z_{i1U}}\right)}^{\prime }$ for k=2 and with k=1 as the reference category.

The transition probabilities describe the probabilities of staying in the attentive or C/IER state or of transitioning to the respective other state. Hence, they indicate the probabilities of being in state k at timepoint t>1 conditional on state l∈{1,2} at t−1. Note that the regular so‐called discrete‐time (DT) LMM assumes the intervals between measurements, $\delta_{ti}$, are equal. In contrast, the intervals can differ across timepoints and individuals in the so‐called continuous‐time (CT) LMM (Böckenholt, 2005; Jackson & Sharples, 2002; Vogelsmeier, Vermunt, Böing‐Messing, & De Roover, 2019). Because differences in intervals are more realistic in EMA (and the CT LMM generalizes to the DT LMM if intervals are equal), in this article, only the CT LMM is applied and described (for a detailed description of the DT LMM, we refer the reader to Vogelsmeier, Vermunt, van Roekel, and De Roover, 2019).

The transition probabilities $p_{\delta_{ti},lk}=p_{\delta_{ti}}(s_{itk}=1|s_{it-1,l}=1,z_{it})$ are collected in the 2×2 matrix $P_{\delta_{ti}}$, where the row sums of $P_{\delta_{ti}}$, $\sum_{k=1}^{2}p_{\delta_{ti},lk}$, are equal to 1. In the CT LMM, the transition probabilities $P_{\delta_{ti}}$ are a function of the interval $\delta_{ti}$ and the “transition intensity matrix” **Q**. The 2×2 matrix Q contains the transition intensities (or rates) $q_{lk}$ defining the transitions from the origin state l to the destination state k measured in a very small time unit. The intensities for the two off‐diagonal elements in the matrix **Q** (i.e., k≠l) are

(7) $$q_{lk}=\underset{\delta \to 0}{\lim}\left(\frac{p(s_{itk}=1|s_{it-\delta ,l}=1,z_{it})}{\delta }\right),$$

and the two diagonal elements are equal to $-\;\sum_{k\neq l}q_{lk}$ (Cox & Miller, 1965). Taking the matrix exponential of $Q\times \delta_{ti}$ results in the transition probabilities $P_{\delta_{ti}}$, implying that the probability of transitioning to the respective other state instead of staying in either the attentive or C/IER state at two consecutive measurement occasions (i.e., k≠l) is more likely for longer intervals. As can be seen from Equation (7), like the initial state probabilities, the transition intensities (and, in turn, the transition probabilities) can depend on covariates, $z_{it}$. Typically, a log‐linear model for the transition intensities is used (again for k≠l):

(8) $$\log q_{lk}=\gamma_{0lk}+\gamma_{lk}^{\prime }z_{it},$$

with $\gamma_{0lk}$ as transition intercepts and $\gamma_{lk}^{\prime }={\left(\gamma_{lk,Z_{i11}},\text{…},\gamma_{lk,Z_{i1U}}\right)}^{\prime }$ as transition slopes that quantify the covariate effects on transitioning compared to staying.

### 3.4 Estimation

The parameters of the proposed model are obtained via maximum likelihood estimation. We chose a step‐wise estimation approach as recommended by Vogelsmeier, Vermunt, Bülow, and De Roover (2021). The three steps are (1) estimating the state‐specific measurement models, (2) assigning observations to the most probable state based on the posterior state‐membership probabilities and calculating classification error inherent to the modal assignment, and (3) evaluating the transition model (including covariate effects). In this third step, the state‐specific measurement model parameter estimates from step 1 remain fixed, and the classification error from step 2 is taken into account. Specifically, the state assignments from step 2 are treated as manifest indicators (containing error) of the true (error‐free) latent states. Equating the error of the manifest indicators to the error computed in step 2 has been shown to provide unbiased parameter estimates and standard errors. For technical details and a simulation study, see Vogelsmeier, Vermunt, Bülow, and De Roover (2021). To assess which covariates are significantly related to the transition model parameters (and, thus, whether they should be included in the model), one may employ backward selection utilizing Wald tests (Agresti, 1990), which is also the chosen approach for this study.

The step‐wise approach is especially favourable when investigating covariate effects on transitions between states, as only the transition model requires re‐estimation (i.e., step 3) when adding or removing covariates, while the estimates for the measurement models remain the same. Additionally, the step‐wise estimation can be performed using the open‐source software R (R Core Team, 2021), while the alternative one‐step full information maximum likelihood estimation (e.g., Vogelsmeier, Vermunt, Böing‐Messing, & De Roover, 2019) requires proprietary software like Latent GOLD (Vermunt & Magidson, 2021), which we see as a notable drawback in terms of moving open science forward. More specifically, step 1 can be performed using the R package mirt (Chalmers, 2012), step 2 can be manually coded, and step 3 can be conducted utilizing the R package lmfa (Vogelsmeier & De Roover, 2021). The syntax for the following empirical application is provided at the Open Science Framework (OSF) (https://osf.io/uj6sr/).

## 4 EMPIRICAL APPLICATION

The EMA data that we use to illustrate the proposed methodology stem from a previous study, which presented novel strategies for assessing the psychometric properties of EMA questionnaires using a “momentary satisfaction with life” scale (Rein et al., 2022). We applied the novel model‐based C/IER detection approach to get insight into (1) how many and which observations are classified into the C/IER and attentive latent states respectively, (2) how individuals transition between the latent states over time, and (3) potential situation‐/participant‐level predictors of transitions among the latent states. Note that no analyses or hypotheses were preregistered beforehand. The primary objective of this application is to showcase the implementation and interpretation of the novel methodology, as well as to explore potential predictors associated with transitioning to the latent C/IER state. Therefore, any conclusions should be validated in future research. The secondary data analysis was approved by the School of Social and Behavioral Sciences Ethics Review Board of Tilburg University, The Netherlands (reference number: RP_FT29). In what follows, we describe the study‐design characteristics, participant information, and measures most relevant to this empirical application. For a complete description, we refer to the original study.

### 4.1 Study design and participants

Participation in the study took 15 days. The study consisted of three parts: an introductory survey on day 1, a 14‐day EMA on days 2 to 15, and a final survey on the evening of day 15. The study was implemented in m‐path (www.m‐path.io). Upon signing up, participants received an information letter and gave their informed consent. As the EMA could impose a burden on the participants, they were informed that participation was voluntary and that they could withdraw from the study at any time. As a token of appreciation for participating in the study, participants were offered the opportunity to receive personalized feedback and to participate in a raffle for Amazon vouchers. It is important to highlight that the participants' chances of winning a voucher was higher if they filled in more questionnaires (i.e., with a higher compliance). This may have incentivized participants to engage in C/IER rather than to omit questionnaires when they were not fully motivated at a given measurement occasion.

After consenting to participate in the study, the participants completed the introductory survey, which comprised demographic variables and questions concerning the study participation (e.g., whether participants wished to receive personalized feedback). On the next morning, the EMA began, which employed a signal‐contingent sampling design (Scollon et al., 2009) with six prompts per day (i.e., up to 84 EMA surveys per participant in total). Participants were notified once randomly within 2‐h blocks (e.g., the first notification was sent between 8 and 10 a.m., the second between 10 a.m. and 12 p.m., and so on). These surveys comprised items on momentary satisfaction and, on some days, instructional manipulation check items. To reduce the time needed for filling in the surveys and thus reduce participant burden, a planned missingness design (Silvia et al., 2014) was used.^4^ The median time required to finish the EMA surveys was 37 s. On the evening of the 15th day, participants were asked to fill out the final survey, which concluded their participation in the study. The final survey assessed the participants' study experience.

The data comprised 71 participants between 18 and 49 years old (M = 25.1, SD = 7.38), of which 24 identified as male and 46 as female (one did not disclose their gender). On average, participants filled in 61.1 out of 84 EMA surveys (≈ 72.7% compliance rate, 4335 observations total).

### 4.2 Measures

In what follows, we describe two types of measures: time‐varying (i.e., situation‐level) measures from the EMA surveys and time‐constant (i.e., person‐level) measures from the final survey.

#### 4.2.1 EMA surveys

Participants received seven out of 10 items measuring the unidimensional construct “momentary satisfaction” at every measurement occasion. Specifically, participants were shown a consistent anchor item along with a randomized selection of six items from the remaining nine. For each item, the participants evaluated their answer on a Likert scale ranging from 0 (strongly disagree) to 6 (strongly agree). The scale was shown to have a high reliability across both time when averaging across persons (r=0.91) and across persons when averaging across time (r=0.99). The items, along with their abbreviations, are provided in Table 1. In what follows, these 10 items are referred to as the *content items*.

**TABLE 1:** Momentary satisfaction questionnaire.

Item	Abbreviation
1. In this moment, I consider myself happy.	consider_happy
2. My current activity makes me satisfied with life.	satisfied_life
3. I would like to change many things about my current situation.*	change_many
4. In this moment, I am experiencing life close to my ideal.	life_ideal
5. My current activity makes me happy	makes happy
6. All things considered, I am satisfied with my current situation.	satisfied_situation
7. If I could relive this moment, I would change nothing.	change_nothing
8. My current activity leaves a lot to be desired.*	leaves_desired
9. I get the important things that I want in life from my current situation.	important_things
10. This moment is in line with how I want my life to be.	line_life

*Note*: An asterisk denotes negatively worded items. Item 10 is the anchor item presented at every measurement occasion. Instructions for the scale are as follows: “Please indicate how much the statements describe your feelings and experiences in the moment right before you received this notification. Please answer honestly and spontaneously. There are no correct or incorrect answers.”

Additionally, participants received instructional manipulation check items at specific measurement occasions to monitor signs of careless responding. Each participant was shown one such item on days 4, 7, 10, and 13 (i.e., at four of 84 measurement occasions). More specifically, the participants were asked to select one specific answer option, for example, “Please select ‘disagree’ for this question.” For this application, 18 participants who specified the wrong answer at least once during participation were considered careless. The other 53 participants were considered attentive. In what follows, this dichotomous participant‐level measure is referred to as *CR_check* (careless responders according to check) with the two categories 1 (yes) and 0 (no).

In addition to the questionnaire‐based measures, two situation‐level measures were considered for each measurement occasio.: First, the number of the current observation, *n_obs*, was computed for each participant and occasion, counting only the completed questionnaires while excluding skipped ones. This metric ranges from 1 to the total number of observations recorded per participant. Second, the hour of the day, *hour_day*, was derived from the submission time of the questionnaires. This metric ranges from 8 to 21, as questionnaires were exclusively sent during this timeframe.

#### 4.2.2 Final survey

After the final EMA questionnaire, participants were asked two questions about their study experience: (1) “If you were to participate in a similar study again, would you prefer a different study length (fewer or more days)?” and (2) “If you were to participate in a similar study again, would you prefer a different number of surveys per day?” For both questions, participants were given the answer options 1 = “yes, fewer [days/surveys per day]”, 2 = “yes, more [days/surveys per day]”, and 3 = “no”. For this application, participants who indicated option 1 were considered overly burdened by the study length and/or the number of surveys per day. This applied to 51 and 34 participants respectively. Participants who indicated option 2 or 3 were not considered overly burdened, which applied to the remaining 16 and 33 participants respectively. In what follows, these two participant‐level predictors are referred to as *burdened_sl* (burdened by the study length) and *burdened_sf* (burdened by sampling frequency) with the two categories 1 (yes) and 0 (no) respectively.

In summary, the data comprised 10 content items, three participant‐level measures (*CR_check*, *burdened_sl*, and *burdened_sf*) and two situation‐level measures (*n_obs* and *hour_day*). The content items were utilized as indicators of the unidimensional construct “momentary satisfaction” in step 1. All other measures were considered potential predictors of latent state transitions in step 3.^5^

### 4.3 Analysis strategy

Our analysis adhered to the three consecutive steps outlined in the Estimation section. In step 1, we analysed the raw content item response data with the confirmatory mixture IRT model for identifying C/IER described earlier. We assumed a unidimensional model held for attentive item responses. We ran the model with 50 sets of random starting values. The solution with the largest log‐likelihood was replicated in roughly 40% of replications. Next, in step 2, the observations were assigned to either the attentive or the C/IER state based on their most probable state membership. Furthermore, the classification error was calculated. Finally, in step 3, the transition model was estimated to investigate the transitions between the attentive and C/IER states, and the effects of five predictors on the transition probabilities were explored. From the variables available in the data, we chose the three participant‐level measures *CR_check*, *burdened_sl*, and *burdened_sf* and the two situation‐level measures *n_obs* and *hour_day* as potential predictors of transitioning to the C/IER state.

First, instructional manipulation check items can act as a preliminary gauge of participants' overall attentiveness (Curran, 2016; Meade & Craig, 2012). Therefore, we expected that participants identified as careless through these checks (*CR_check* = 1) would show higher probabilities of transitioning to and remaining in the C/IER state than participants identified as attentive (*CR_check* = 0).

Second, participant burden is expected to reduce attentiveness s (Hasselhorn et al., 2023; Ulitzsch et al., 2024). The data for this application encompass two distinct forms of burden: one related to the duration of the study and the other associated with the sampling frequency. While the relation between study length and C/IER remains largely unexplored, researchers experimentally manipulated sampling frequencies in EMA studies and did not find significant effects on C/IER (Eisele et al., 2022; Hasselhorn et al., 2023). Nevertheless, in this application, we included both perceived burden due to study length and due to sampling frequency as predictors of transition probabilities. Assessing (predictors of) C/IER is still a newly emerging stream of research, and it remains important to investigate the effects of design choices in different studies, especially when using differing measures of sampling frequency (burden) and novel methodologies to identify C/IER. We expected participants who were burdened by either the study length (*burdened_sl* = 1) or the sampling frequency (*burdened_sf* = 1) to have higher probabilities of transitioning to and staying in the C/IER state than participants who were not burdened (*burdened_sl* = 0 and *burdened_sf* = 0).

Finally, attentiveness can generally decrease over time – throughout the course of participation in general but also intraday – as a result of growing bored with repeatedly completing the same questionnaires (i.e., fatigue effects; Eisele et al., 2023). Attentiveness within a day may additionally decline because of reduced cognitive skills throughout the day (Schmidt et al., 2007; West et al., 2022). Previous research indeed indicated a decline in attentiveness with study duration (Denison, 2022; Ulitzsch et al., 2024), but discernible within‐day trends did not emerge (Ulitzsch et al., 2024). However, the effect of time of day on C/IER is still largely unexplored in EMA research. In this application, we included *hour_day* and *n_obs* as predictors of the transition probabilities, where the latter served as a proxy for elapsed time of participation. We expected higher probabilities of transitioning to and staying in the C/IER state for larger values on both predictors than for lower ones.

For the predictor selection, we used backward selection; that is, we started with a transition model including all predictors and removed them one by one until only those with significant Wald‐test statistics were in the model.^6^ After selecting the model, we first examined the transition probabilities for a 1‐h interval^7^ and predictor values corresponding to their sample means to obtain an overall impression of the stability of state memberships. Subsequently, for each predictor, we compared the transition probabilities for a 1‐h interval for the lowest score in the sample to the probabilities for the highest score, keeping the predictor values for the other two predictors equal to their sample means.^8^For the categorical predictors, the lowest and highest values simply refer to the meanings of the values 0 and 1.

The analyses were performed in R (R Core Team, 2021, R version 4.2.3). Step 1 was performed using the R package mirt version 1.41 (Chalmers, 2012), step 2 was manually coded, and step 3 was conducted utilizing the R package lmfa version 0.1.3 (Vogelsmeier & De Roover, 2021).^9^ The data and the code for the data analysis are publicly available at OSF (https://osf.io/uj6sr/).

### 4.4 Results

#### 4.4.1 Measurement model

Approximately 8% of the observations were assigned to the C/IER state. The classification‐error probabilities were close to zero for the attentive state (0.01) but considerably higher for the C/IER state (0.29). This implies that the classification of observations assigned to the C/IER state was relatively less certain compared to the classification of observations assigned to the attentive state.

**FIGURE 2:**
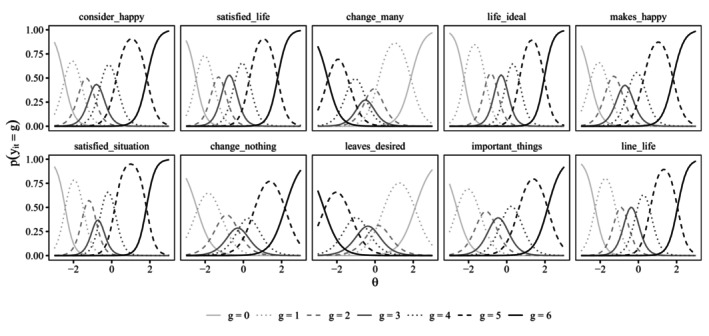
Item characteristic curves in attentive state for the 10 content items. θ: momentary satisfaction.

**FIGURE 3:**
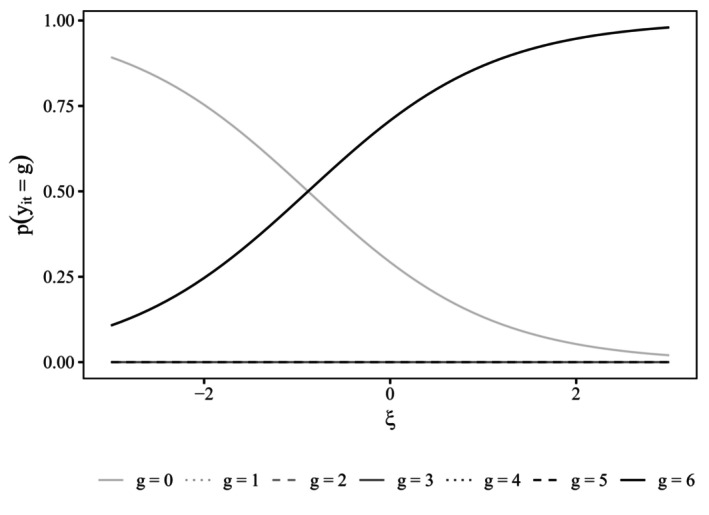
Item characteristic curve for C/IER state. ξ: category preference.

Figures 2 and 3 display item characteristic curves (ICCs) for the attentive and C/IER states. Recall that for the C/IER state, item parameters are constrained to be the same across items and, hence, only a single ICC is obtained. The x‐axis range was chosen to align with the estimated variance of the latent category preference factor of 0.997, displaying the ICC for the latent category preference continuum within ± three standard deviations around the mean.

As was to be expected, in the attentive state, for the positively worded items, the probability of choosing a higher response category increased with a higher location on the momentary satisfaction trait but decreased for negatively worded items. In the C/IER state, category probabilities for the middle categories (1–5) were essentially zero across the latent category preference continuum. Instead, when in the C/IER state, participants tended to favour one of the extreme categories (0 or 6).

#### 4.4.2 Transition model

Starting from the model with all predictors included, we first removed *burdened_sl* and then *n_obs* because the Wald‐test statistics were non‐significant. Thus, contrary to our expectations, these predictors were unrelated to the transitions between the attentive and the C/IER state. In line with our expectations, the remaining three predictors were significantly related to the transition probabilities (*CR_check*: $\chi^{2}(2)=27.45$, p<0.001; *hour_day*: $\chi^{2}(2)=6.57$, p=0.04; *burdened_sf*: $\chi^{2}(2)=12.75$, p<0.001).

Investigating the transition probabilities for a 1‐h interval and predictor values corresponding to their sample means (i.e., 0.29 for *CR_check*, 13.81 for *hour_day*, and 0.51 for *burdened_sf*) indicated that the staying probabilities were equal to 0.98 and 0.82 for the attentive and C/IER states respectively. Hence, the probabilities of transitioning from the attentive to the C/IER state and in the reverse direction were 0.02 and 0.18 respectively. Therefore, staying in a state was generally more likely than transitioning away from it. However, the stability was more pronounced in the attentive state. In line with this, 15 of the 71 individuals consistently remained in the attentive state. None of the participants consistently stayed in the C/IER state.

Comparisons of the transition probabilities for the lowest and highest predictor values are shown in Table 2, and the results for each predictor are described below.

**TABLE 2:** Transition probabilities for low and high values on the three predictors while holding all other predictor values equal to their sample means.

Predictor (low value → high value)	From/To	Attentive		C/IER	
*CR_check* (attentive → careless)	Attentive	0.99 → 0.96	(−)	0.01 → 0.04	(+)
	C/IER	0.18 → 0.18	( )	0.82 → 0.82	( )
*hour_day* (8 h → 21 h)	Attentive	0.99 → 0.96	(−)	0.01 → 0.04	(+)
	C/IER	0.10 → 0.35	(+)	0.90 → 0.65	(−)
*burdened_sf* (no → yes)	Attentive	0.96 → 0.99	(+)	0.04 → 0.01	(−)
	C/IER	0.37 → 0.09	(−)	0.63 → 0.91	(+)

*Note*: The interval length for which the transition probabilities were compared was equal to 1 h. The value to the left of the arrow represents the transition probability associated with the lowest (categorical or continuous) predictor value in the sample while holding all other predictor scores equal to their sample means; the value to the right of the arrow indicates the transition probability associated with the highest predictor value in the sample while holding all other predictor scores equal to their sample means; the (meanings of) the lowest and highest values for each predictor are provided in the first column. The sign in parentheses indicates the increment/decrement between the two values; the parentheses are empty if the value does not differ. The cell colour is green if the transition probability rises with an increase in the predictor value, red if it decreases, and white if it stays the same. The strength of the colour indicates the magnitude of the change.

##### CR_check

Comparing the transition probabilities for participants who were flagged as attentive according to the instructional manipulation check items to the probabilities for participants who were flagged as careless showed that, in line with our expectations, the probability of transitioning to the C/IER state was slightly higher for the careless participants (0.04) than for the attentive participants (0.01). However, the probabilities of staying in the C/IER state were the same for careless and attentive participants (0.82). Overall, this indicates that the predictor is practically rather uninformative for the probabilities of participants being in the C/IER state, although it was significantly related to the transition probabilities. This is likely the result of an artificially inflated sample size for the participant‐level predictors, which is explained in more detail in the Discussion section.

##### Hour_day

The comparison of the transition probabilities when completing the EMA questionnaires at 8 a.m. with the probabilities when completing the questionnaires at 9 p.m. showed that the probabilities of transitioning to the C/IER state were only slightly higher in the evening (0.04) than in the morning (0.01) and that the probabilities of remaining in the C/IER state were considerably lower in the evening (0.65) than in the morning (0.90). Contrary to our expectation, this indicates that it is generally less likely to be in the C/IER state in the evening than in the morning.

##### Burdened_sf

Comparing the transition probabilities for participants who were not burdened by the sampling frequency with the probabilities of participants who were burdened, we found that the probability of transitioning to the C/IER state was slightly lower for burdened participants (0.01) than for non‐burdened participants (0.04) but, more importantly, that the probability of remaining in the C/IER state was significantly higher for burdened participants (0.91) than for non‐burdened participants (0.63). Overall, this shows that, in line with our expectations, experienced burden regarding the sampling frequency is related to a higher probability of being in the C/IER state.

### 4.5 Conclusions

Approximately 8% of observations were classified as stemming from C/IER. This is a noteworthy amount considering that the data were collected using a planned missingness design to reduce participant burden by shortening the questionnaire because a shorter questionnaire length was shown to be associated with more attentive responding than long questionnaires in EMA (Eisele et al., 2022). This highlights the need to model and understand C/IER in EMA data, even under seemingly favourable study designs.

The overall within‐person stability of attentive responding and C/IER was high. However, situation‐level predictors altered the probabilities of engaging in both types of responding. Specifically, the perceived burden due to sampling frequency and participation at earlier times of the day were related to a higher probability of C/IER. The latter finding did not align with our initial expectations. A possible explanation is that the time of day serves as more than just a timestamp; it might also reflect the location of the participants and the time available to complete the EMA attentively. Considering the overall young age of the participants, it is plausible that they have greater mental capacity to respond attentively in the later hours of the day, after their study or work commitments have ended. However, further research, including experimental manipulation, is needed to better understand the effects of time of day on C/IER in EMA. Likewise, future research may explore more complex (e.g., quadratic) relationships between time of day and C/IER.

## 5 EVALUATING ROBUSTNESS AGAINST TYPICAL CHALLENGES ENCOUNTERED IN ECOLOGICAL MOMENTARY ASSESSMENTS

We probed the trustworthiness of C/IER detection with the proposed approach against typical challenges encountered in EMA data in three simulation studies. First, typical EMA questionnaires may exhibit properties that entail low separability of attentive and inattentive item responses. This pertains to, firstly, the homogeneity of the administered items and, second, to sparse usage of negatively worded items. In particular, homogeneous EMA questionnaires without negatively worded items are much more probable to elicit attentive item response patterns closely resembling straightlining behaviour and/or inattentive response behaviour with strong person‐specific preferences for some categories. Therefore, in Study I, we explored the impact of varying EMA questionnaire properties on C/IER detection under different types of C/IER behaviour, assuming that homogeneous, purely positively worded EMA questionnaires may pose a particular challenge under the boundary condition of all inattentive respondents displaying straightlining behaviour.

Secondly, the proposed method rests on the assumption that the attentive and C/IER mixture component model parameters generalize across all observations. For trustworthy C/IER detection, we believe that this assumption is less of an issue for the C/IER component model. The C/IER component model by Uglanova et al. (in preparation) is very capable of handling multiple types of C/IER behaviour across observations, occurring, for instance, when some respondents select their careless responses randomly across the board, while others tend to opt for some specific categories. Unaccounted for violations of measurement invariance for the attentive component model across individuals and/or time, however, may entail artefactual conclusions on C/IER rates. This is especially problematic because such violations are plausible (Adolf et al., 2014; Brose et al., 2015; McNeish et al., 2021; Vogelsmeier, Cloos, et al., 2023; Vogelsmeier, Vermunt, van Roekel, & De Roover, 2019): Because participants fill out their questionnaires in various contexts and situations, the structure of measurement may vary across the course of an EMA study.

Studies II and III served to determine the degree of violations that still allowed for obtaining trustworthy conclusions on C/IER occurrence. Study II evaluated the robustness of C/IER detection against unaccounted for heterogeneity in attentive loading patterns across observations. Thus, Study II mimicked a scenario of reprioritization in attentive responding (Oort et al., 2005), where single items changed in their importance for the measured constructs.

Study III investigated the consequences of unaccounted for heterogeneity in factor structure across observations. Such heterogeneity occurs when the nature of the measured constructs changes across the course of the EMA. For instance, when responding to items assessing affective experiences, respondents may shift from a valence to an arousal focus in some contexts, causing the underlying factor structure to shift from positive affect and negative affect factors to high and low arousal factors (Feldman, 1995). Likewise, dampened emotional granularity (i.e., lower differentiation between emotions) can be assumed to result in increased correlations among emotional facets (Krone et al., 2018), with the most extreme scenario being that emotional facets collapse into a single factor for observations with low granularity.

In our evaluations, we focused on the trustworthiness of C/IER detection in step 1 of the proposed approach. This is a key prerequisite for accurate conclusions drawn based on the subsequent steps 2 and 3, which serve to get a better understanding of respondent and occasion characteristics associated with the occurrence of (presumed) C/IER.

### 5.1 Methods

To evaluate C/IER detection under realistic research conditions, we mimicked the EMA study of the empirical application reported above. We generated data for I=75 respondents being administered J=10 7‐point Likert‐scale items at T=60 measurement occasions. Data for each observation (i.e., respondent‐by‐occasion interaction) were generated independently.^10^

In all simulation conditions, 10% of the observations were simulated to stem from C/IER.^11^ To simulate C/IE responses, we followed recommendations by Curran and Denison (2019) and simulated different C/IE response patterns. In Study I, patterns were varied to study how their presence might impair or alleviate C/IER detection under different questionnaire conditions. In Studies II and III, we considered a (presumably realistic) mix of different types of behaviour. Doing so allows us to showcase that the proposed approach can indeed deal with the simultaneous occurrence of different C/IER response patterns. C/IER observations in Studies II and III were randomly assigned to two groups. For the first group of purely random responders, C/IER responses were generated uniform randomly. For the second group of responders with category preferences, C/IER responses were generated according to the constrained GRM component model given in Equation (3), with loadings and thresholds set to the values obtained in the empirical application (Table A1). C/IER category preference traits were drawn from a standard normal distribution.

We simulated attentive responses according to a unidimensional GRM, employing the loadings and thresholds obtained for the attentive component model in the empirical application (Table A2) and drawing respondents' traits from a standard normal distribution. Recall that the items in the empirical application were already rather homogeneous but contained two negatively worded items. Hence, in Study I, we manipulated the data‐generating parameters for the attentive component model by (a) increasing item heterogeneity and (b) assuming positively worded items only. In Studies II and III, in the baseline condition, all attentive item responses were generated from the same model according to the parameters obtained in the empirical application. To study robustness, the attentive measurement model was manipulated for some observations, inducing heterogeneity in loading patterns (Study II) or factor structures (Study III).

For each simulation condition, we generated 50 data sets. In line with step 1 of the proposed approach, each data set was analysed with the confirmatory mixture IRT model proposed by Uglanova et al. (in preparation), Equations (1) and (3). treating all repeated observations as independent, using the R package mirt version 1.41 (Chalmers, 2012). For each replication, we ran the model with 10 sets of random starting values and selected the solution with the largest log‐likelihood. To evaluate convergence, we evaluated whether the solution with the largest log‐likelihood met the mirt default convergence criteria after a maximum of 1000 iterations.

As evaluation criteria, we considered the convergence, bias, and variability of C/IER rate estimates as well as sensitivity (i.e., the proportion of correctly identified C/IER observations out of all observations classified as C/IER) and specificity (i.e., the proportion of correctly identified attentive observations out of all observations classified as attentive) of observation‐level C/IER classification using modal assignment. Further, we inspected mean posterior C/IER state probabilities for true positive, true negative, false positive, and false negative C/IER observations to gauge the classification error for these types of observations. All analyses were conducted using R (R Core Team, 2021, R version 4.2.3).

#### 5.1.1 Study I: Investigating separability under varying questionnaire conditions

**FIGURE 4:**
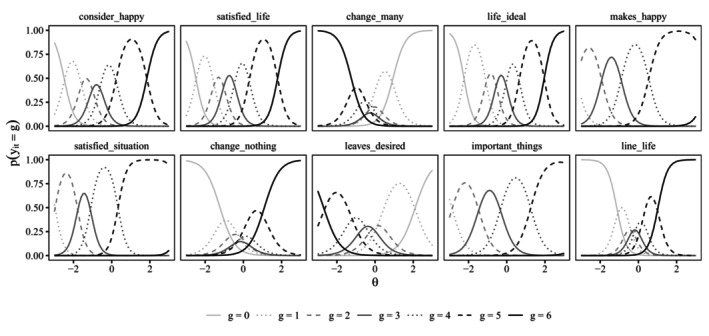
Exemplar data‐generating item characteristic curves in the attentive state for the 10 content items altered to increase item heterogeneity. θ, momentary satisfaction.

In Study I, we manipulated EMA questionnaire characteristics in terms of (a) item heterogeneity and (b) the presence of negatively worded items. In low item heterogeneity conditions, we employed the item thresholds obtained in the empirical application. In high item heterogeneity conditions, item thresholds of two sets of three randomly selected items were multiplied by 0.50 and 2.00 respectively, while item thresholds for the remaining four items remained unaltered. Note that we deliberately chose such extreme alterations for illustrative purposes. Figure 4 depicts an example of resultant ICCs in high heterogeneity conditions. In conditions without negatively worded items, we took the absolute values of all item loadings obtained in the empirical application. In conditions with negatively worded items, the loadings of two randomly selected items were set to be negative. For generating C/IER response patterns, we considered three extreme conditions. In the first condition, all C/IER respondents engaged in uniform random responding. In the second condition, we considered a strong preference for the midpoint, where, for all C/IER respondents, the probability of selecting was set to 0.80, and the probabilities for the two adjacent and remaining categories were set to 0.05 and 0.025 respectively. This condition may challenge C/IER detection as inconsistencies in agreeing or disagreeing to differently worded items cannot be exploited.^12^ In the third condition, all C/IER respondents were simulated to exhibit straightlining, with the respondent‐specific selected value being determined randomly. According to simulation results reported in Uglanova et al. (in preparation), this is the pattern that challenges C/IER detection with the proposed approach the most and, therefore, aids in exploring boundary conditions of the proposed approach. First, as opposed to strongly pronounced category preferences where selected categories still probabilistically vary given the respondent, the selection of subsequent categories is deterministic under straightlining behaviour given the respondent, violating basic assumptions of IRT. Second, straight lining will be accommodated by the utilized component model with very large estimates of the latent category preference variance, with values often being far beyond the typical parameter space considered in default random starting values, which may lead to convergence issues or convergence to local maxima and biased parameter estimates.

#### 5.1.2 Study II: Heterogeneity in loading patterns

**FIGURE 5:**
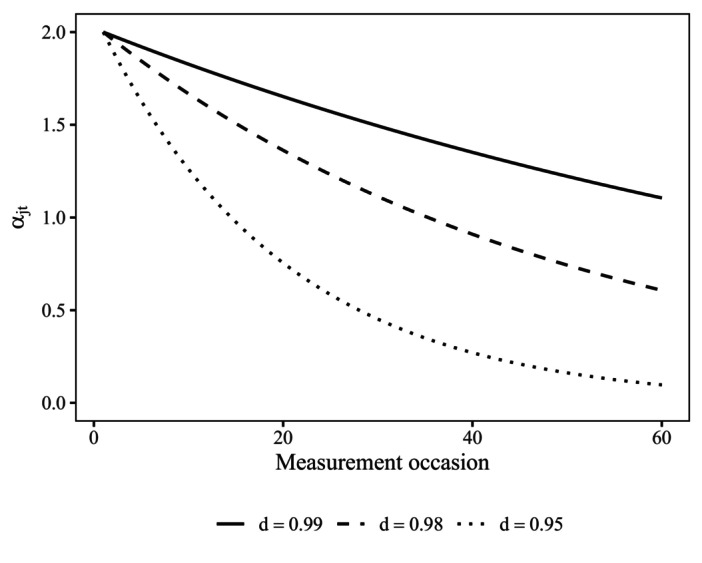
Change in item loadings across measurement occasions for different severity of exponential decay d.

In Study II, we evaluated the effects of changing loading patterns, while the overall factor structure remained intact across attentive respondents and measurement occasions. We randomly selected a varying number of items (2;5;10) that were affected by an exponential decay in their loadings across measurement occasions, assuming that more change occurs at the beginning of the study.^13^ Then, for the affected items, we gradually decreased loadings across measurement occasions as $\alpha_{jt}=\alpha_{j1}\cdot d^{\left(t-1\right)}$, varying the severity of exponential decay d (0.99;0.98;0.95). Figure 5 illustrates the trajectory of three item loadings with $\alpha_{j1}=2$ and exponential decay of d=0.99, d=0.98, and 0.95 across T=60 measurement occasions. As can be seen, for d=0.99, original item loadings were approximately halved by the last measurement occasion. In the most extreme condition of d=0.95, item loadings of affected items were essentially zero at the last measurement occasion.

#### 5.1.3 Study III: Heterogeneity in factor structure

In Study III, we evaluated the effect of changes in attentive factor structure across the EMA. We randomly selected a varying proportion of observations (0.20;0.30;0.50) for which a two‐dimensional instead of a unidimensional attentive measurement model with varying latent correlation ρ (0.80;0.60;0.40) was simulated. For the two‐dimensional attentive measurement model, the first five items measured the first and the remaining items the second factor. Data‐generating values for loadings and thresholds were the same as for the unidimensional model.

### 5.2 Results

#### 5.2.1 Study I: Investigating separability under varying questionnaire conditions

Table 3 displays convergence rates and quality of C/IER identification for Study I. In all conditions where respondents exhibited uniform random responding or straightlining, convergence rates were above 0.90. In conditions with strong midpoint preferences, the estimation was challenged, interestingly, even more so when item heterogeneity was high and negatively worded items were included. In this condition, only 68% of the replications converged.

**TABLE 3:** Convergence rates and quality of C/IER identification for different conditions of heterogeneity in attentive loading patterns in Study I.

H	$J_{n}$	Pat	Conv	Bias $\pi_{2}$	SD $\pi_{2}$	Sens	Spec	${\bar{\pi_{2}}}^{\text{TP}}$	${\bar{\pi_{2}}}^{\text{TN}}$	${\bar{\pi_{2}}}^{\text{FP}}$	${\bar{\pi_{2}}}^{\text{FN}}$
Low	0	URR	0.92	0.001	0.005	0.997	0.988	0.991	0.003	0.714	0.172
		MP	0.86	−0.000	0.005	0.998	0.984	0.988	0.001	0.769	0.227
		SL	1.00	−0.068	0.016	0.933	0.954	0.990	0.001	0.829	0.001
	2	URR	0.90	0.000	0.004	0.997	0.991	0.992	0.002	0.733	0.175
		MP	0.76	0.002	0.005	0.999	0.991	0.993	0.001	0.758	0.242
		SL	1.00	−0.019	0.008	0.977	0.984	0.997	0.001	0.764	0.000
High	0	URR	0.90	−0.000	0.004	0.998	0.991	0.993	0.002	0.712	0.168
		MP	0.80	−0.001	0.004	0.999	0.994	0.995	0.001	0.793	0.227
		SL	0.99	−0.031	0.011	0.962	0.963	0.997	0.003	0.762	0.000
	2	URR	0.94	−0.001	0.005	0.998	0.992	0.994	0.002	0.716	0.162
		MP	0.68	−0.001	0.005	0.999	0.995	0.997	0.000	0.765	0.240
		SL	0.92	−0.015	0.005	0.982	0.996	1.000	0.000	0.771	0.000

Abbreviations: Bias $\pi_{2}$, bias of C/IER mixture proportion estimate; H, item heterogeneity; $J_{n}$, number of negatively worded items; MP, preference for midpoint; Pat, C/IER response patterns; SD $\pi_{2}$, standard deviation of C/IER mixture proportion estimate across replications; Sens, sensitivity of observation‐level C/IER classification using modal assignment; Spec, specificity of observation‐level C/IER classification using modal assignment; SL, straightlining; convergence: proportion of converged replications; URR, uniform random responding; ${\bar{\pi_{2}}}^{\text{TP}}$, mean posterior C/IER state probability for true C/IER observations classified as C/IER; ${\bar{\pi_{2}}}^{\text{TN}}$, mean posterior C/IER state probability for truly attentive observations classified as attentive; ${\bar{\pi_{2}}}^{\text{FP}}$, mean posterior C/IER state probability for truly attentive observations classified as C/IER; ${\bar{\pi_{2}}}^{\text{FN}}$, mean posterior C/IER state probability for true C/IER observations classified as attentive.

When C/IER respondents solely engaged in uniform random responding, C/IER rate estimates were unbiased and exhibited low variability, while sensitivity and specificity were very high across all conditions. Likewise, despite the encountered convergence issues, parameters under strong midpoint preferences were well recovered. For both patterns, mean posterior C/IER state probabilities for true positive (i.e., true C/IER observations classified as C/IER with modal assignment) and true negative (i.e., truly attentive observations classified as attentive) C/IER observations were close to 1 and 0 respectively, indicating very low classification error for these observations. For false positive (i.e., truly attentive observations classified as C/IER) and false negative (i.e., true C/IER observations classified as attentive) C/IER observations, mean posterior state probabilities were further away from 1 and 0 respectively, indicating that for wrongly classified observations, classification error is higher.

In contrast, in line with results reported in Uglanova et al. (in preparation), the detection of straightlining behaviour was challenged when all items were worded in the same direction, resulting in severe underestimation of C/IER occurrence. This effect was even more pronounced when items exhibited low heterogeneity. Under this condition, bias in the estimated C/IER rate was as high as ‐0.068, that is, on average, the model yielded a C/IER rate of as low as 3.2% as opposed to the data‐generating rate of 10%. At the same time, sensitivity and specificity were somewhat lower, but still high. When questionnaires included negatively worded items, the bias in C/IER rate estimates was markedly alleviated. Bias was sufficiently low so as not to drastically alter conclusions on C/IER occurrence when the inclusion of negatively worded items was further paired with high item heterogeneity. Under this condition, on average, the model yielded a C/IER rate of 8.5% as opposed to the data‐generating rate of 10%. Note that the inclusion of (even only a few) negatively worded items showed to be much more decisive for trustworthy C/IER detection than items exhibiting a very high degree of heterogeneity. Interestingly, mean posterior C/IER state probabilities for false negative C/IER observations was close to 0 under conditions with straightlining behaviour; that is, classification error tended to reflect misclassification to a lesser extent.

These results indicate that the employed confirmatory mixture model is indeed capable of trustworthy detection of different C/IER patterns under typical EMA conditions of low heterogeneity and uniform polarity of the administered items. Pure straightlining poses a boundary condition for the employed confirmatory mixture model. For its detection, we recommend including negatively worded items and, if possible, administering questionnaires with high item heterogeneity.

#### 5.2.2 Study II: Heterogeneity in loading patterns

Table 4 displays convergence rates and quality of C/IER identification for Study II. Conditions with extreme exponential decay (d=0.95) challenged the estimation procedure, resulting in increasing non‐convergence rates with an increasing number of affected items. When all J=10 items were affected, only 82% of the replications converged. Note, however, that this condition is rather extreme and, arguably, not realistic, as it resulted in attentive loadings of essentially zero for all items by the end of the EMA (i.e., none of the items was reflective of the to‐be‐measured trait). In conditions with less extreme exponential decay, all replications converged. For converged replications, the quality of C/IER detection resembled those of the baseline condition without violations of measurement invariance. Bias and variability of C/IER proportion estimates were low, while sensitivity and specificity were high.

**TABLE 4:** Convergence rates and quality of C/IER identification for different conditions of heterogeneity in attentive loading patterns in Study II.

d	$J_{d}$	Conv	Bias $\pi_{2}$	SD $\pi_{2}$	Sens	Spec	${\bar{\pi_{2}}}^{\text{TP}}$	${\bar{\pi_{2}}}^{\text{TN}}$	${\bar{\pi_{2}}}^{\text{FP}}$	${\bar{\pi_{2}}}^{\text{FN}}$
Baseline		1.00	−0.002	0.005	0.992	0.975	0.978	0.005	0.721	0.173
0.99	2	1.00	−0.001	0.004	0.992	0.975	0.977	0.005	0.714	0.177
	5	1.00	−0.003	0.005	0.992	0.976	0.977	0.005	0.719	0.180
	10	1.00	−0.002	0.005	0.992	0.975	0.978	0.005	0.716	0.179
0.98	2	1.00	−0.002	0.005	0.992	0.975	0.977	0.005	0.724	0.181
	5	1.00	−0.001	0.005	0.992	0.973	0.975	0.005	0.717	0.182
	10	1.00	−0.002	0.004	0.992	0.977	0.977	0.005	0.719	0.176
0.95	2	1.00	−0.003	0.005	0.992	0.975	0.976	0.005	0.718	0.181
	5	0.96	0.002	0.005	0.991	0.935	0.977	0.006	0.795	0.179
	10	0.82	−0.000	0.004	0.993	0.962	0.979	0.005	0.799	0.176

*Note*: Bias $\pi_{2}$, bias of C/IER mixture proportion estimate; d, exponential decay; $J_{d}$, number of affected items; SD $\pi_{2}$, standard deviation of C/IER mixture proportion estimate across replications; Sens, sensitivity of observation‐level C/IER classification using modal assignment; Spec, specificity of observation‐level C/IER classification using modal assignment; ${\bar{\pi_{2}}}^{\text{TP}}$, mean posterior C/IER state probability for true C/IER observations classified as C/IER; ${\bar{\pi_{2}}}^{\text{TN}}$, mean posterior C/IER state probability for truly attentive observations classified as attentive; ${\bar{\pi_{2}}}^{\text{FP}}$, mean posterior C/IER state probability for truly attentive observations classified as C/IER; ${\bar{\pi_{2}}}^{\text{FN}}$, mean posterior C/IER state probability for true C/IER observations classified as attentive.

Resembling results for uniform random responding obtained in Study I, mean posterior C/IER state probabilities for true positive and true negative C/IER observations were close to 1 and 0 respectively, indicating very low classification error for these observations. For false positive and false negative C/IER observations, mean posterior state probabilities were further away from 1 and 0 respectively, indicating that for wrongly classified observations, classification error is higher. From these results we conclude that C/IER detection exhibits robustness against the studied types of heterogeneity in attentive loading patterns.

#### 5.2.3 Study III: Heterogeneity in factor structure

Table 5 displays convergence rates and quality of C/IER identification for Study III. No convergence issues were encountered in Study III. However, there was an increasing upward bias in C/IER proportion estimates with an increasing degree of violation of measurement invariance, i.e., with an increasing proportion of observations for which a two‐dimensional instead of the modelled unidimensional attentive measurement model held as well as with a decreasing correlation ρ between the two factors. For highly correlated factors, bias remained minimal, with bias for ρ=0.80 remaining below 1%. For the most extreme condition with 50% of attentive observations being affected and ρ=0.40; however, bias was as high as 0.06. That is, researchers would conclude that 16% instead of the data‐generating 10% of observations stem from C/IER. The increasing upward bias was accompanied by decreasing specificity. Further, mean posterior C/IER state probabilities for false positive C/IER observations approached 1, indicating that for observations wrongly classified as inattentive, the classification error tended to reflect misclassification to a lesser extent than in Study II. From these results we conclude that the approach exhibits robustness only to mild heterogeneity in factor structures.

**TABLE 5:** Convergence rates and quality of C/IER identification for different conditions of heterogeneity in attentive factor structure in Study III.

ρ	Obs	Conv	Bias $\pi_{2}$	SD $\pi_{2}$	Sens	Spec	${\bar{\pi_{2}}}^{\text{TP}}$	${\bar{\pi_{2}}}^{\text{TN}}$	${\bar{\pi_{2}}}^{\text{FP}}$	${\bar{\pi_{2}}}^{\text{FN}}$
Baseline		1.00	−0.002	0.004	0.992	0.975	0.978	0.005	0.723	0.173
0.80	0.20	1.00	0.003	0.005	0.992	0.930	0.975	0.007	0.791	0.181
	0.30	1.00	0.005	0.005	0.991	0.911	0.975	0.008	0.792	0.177
	0.50	1.00	0.008	0.005	0.990	0.886	0.971	0.010	0.794	0.183
0.60	0.20	1.00	0.015	0.005	0.992	0.834	0.976	0.009	0.849	0.185
	0.30	1.00	0.022	0.005	0.991	0.788	0.974	0.011	0.851	0.190
	0.50	1.00	0.034	0.006	0.990	0.718	0.970	0.015	0.845	0.193
0.40	0.20	1.00	0.027	0.005	0.992	0.754	0.976	0.010	0.880	0.187
	0.30	1.00	0.040	0.006	0.992	0.684	0.975	0.013	0.880	0.182
	0.50	1.00	0.062	0.007	0.991	0.595	0.970	0.019	0.877	0.195

Abbreviations: ρ, correlation of latent factors; Obs, proportion of affected observations; Bias $\pi_{2}$, bias of C/IER mixture proportion estimate; SD $\pi_{2}$, standard deviation of C/IER mixture proportion estimate across replications; Sens, sensitivity of observation‐level C/IER classification using modal assignment; Spec, specificity of observation‐level C/IER classification using modal assignment; ${\bar{\pi_{2}}}^{\text{TP}}$, mean posterior C/IER state probability for true C/IER observations classified as C/IER; ${\bar{\pi_{2}}}^{\text{TN}}$, mean posterior C/IER state probability for truly attentive observations classified as attentive; ${\bar{\pi_{2}}}^{\text{FP}}$, mean posterior C/IER state probability for truly attentive observations classified as C/IER; ${\bar{\pi_{2}}}^{\text{FN}}$, mean posterior C/IER state probability for true C/IER observations classified as attentive.

## 6 DISCUSSION

In this study, we introduced a novel method to distinguish between attentive responding and C/IER in EMA while uncovering contextual correlates of C/IER. Our confirmatory mixture modelling approach is suitable for scales with multiple indicators assessed via ordered response categories. It maximizes the leverage of item information by identifying C/IER based on predefined measurement models for attentive and C/IER responses. This eliminates the need for possibly ambiguous post hoc interpretation of latent states resulting from exploratory mixture modelling. As previous mixture modelling approaches for C/IER in EMA data (Hasselhorn et al., 2023; Ulitzsch et al., 2024), it does not require decisions on threshold settings and takes C/IER classification uncertainty into account. Further, because C/IER is identified using content item responses, no attention check items need to be administered, resulting in shorter questionnaires.

Our empirical findings underscore the efficacy of this novel approach in both pinpointing C/IER instances in EMA and gaining insights into their underlying causes. Understanding these reasons is crucial for mitigating C/IER in future studies and thus for optimizing research outcomes. For instance, incorporating additional motivational reminders in situations prone to C/IER occurrence could enhance attentive responding and, in turn, data quality.

We illustrated that the approach was capable of identifying various types of C/IER patterns even under challenging, but typical, EMA conditions of low item heterogeneity and unidirectionally worded items. Nevertheless, to facilitate the detection of straightlining, heterogeneous and negatively worded items are needed. We further evaluated the method's robustness against the unaccounted presence of attentive measurement model changes in two simulation studies. We found the approach to be robust against unaccounted for heterogeneity in attentive loading patterns across observations, but not against unaccounted for heterogeneity in the factor structure underlying attentive responses.

### 6.1 Limitations and future directions

In evaluating the approach, we considered an idealized scenario where each item was administered at all measurement occasions. However, in practice – as in the empirical data used for illustration – EMA researchers often employ planned missingness designs. Future research may focus on C/IER identification in these designs and further explore related challenges, i.e., evaluate C/IER identification under high proportions of missing values and investigate how administering negatively worded items at some, but not all, measurement occasions impacts trustworthy C/IER identification.

Future research may explore methods to accommodate changes in the attentive measurement model. If hypotheses on the type of measurement model changes exist, these can be translated into additional confirmatory measurement models. For instance, researchers may specify multiple attentive states between which correlations among latent factors are allowed to vary to accommodate hypothesized variations in emotional granularity, or they could specify attentive measurement models with positive and negative affect factors and high and low arousal factors respectively. If researchers assume that the perception of a specific item may be context‐dependent, component models with different loading structures could be formulated. For instance, depending on the context, reporting excitement can be reflective of both positive (when associated with enthusiasm) and negative (when associated with nervousness) affect. Likewise, researchers may easily accommodate more complex types of attentive response behaviour. Response styles (i.e., idiosyncrasies in how respondents use rating scales), for instance, recently gained increased attention in EMA research (Deng et al., 2018). These can easily be considered by using an attentive component model that takes response styles into account (see Ulitzsch, Pohl, et al., 2023, for a mixture IRT model accommodating both response styles and C/IER). If no such hypotheses exist, researchers may combine the confirmatory C/IER component model with exploratory attentive measurement models. Such an approach may filter out observations exhibiting C/IER and explore measurement model changes in the remaining attentive observations. Note, however, that such an approach introduces additional complexity due to the need for model selection both in terms of the number and nature of the attentive models. The performance of model selection (e.g., using the Bayesian information criterion as proposed for regular LMFA; Vogelsmeier, Vermunt, van Roekel, & De Roover, 2019) has yet to be evaluated when combining exploratory attentive measurement models with the confirmatory C/IER component model.

Another limitation is that the method is currently suitable only for ordered response options (e.g., resulting from Likert scales). However, the proposed method has the potential to be extended to also accommodate continuous scales (e.g., resulting from visual analog scales). A straightforward approach is to build upon the mixture factor analysis model employed in the original LMFA and specify confirmatory factor models (see Kam & Cheung, 2023, for a constrained factor mixture model for C/IER) instead of exploratory ones within the states (which is possible in Latent GOLD; Vermunt & Magidson, 2021). However, simulation studies are required to evaluate the performance of this extension for distinguishing between attentive responding and C/IER in data gathered using continuous scales. An alternative approach worth exploring is employing a beta item response model accommodating the constrained range of visual analogue scales (Noel & Dauvier, 2007) within the states. However, for beta item response models, C/IER component models still need to be developed.

Obviously, the proposed approach is not applicable when EMAs administer single‐indicator measures, as is often done to keep surveys as short as possible. In this case, the screen‐time‐based mixture modelling approach by Ulitzsch et al. (2024) may be a viable alternative.

To further aid the disentanglement of attentive from C/IER observations, future research may enrich the proposed component models with the additional information on response behaviour contained in item‐level response times. This can easily be achieved by incorporating additional measurement models for attentive and inattentive response times alongside the models for item responses (Ulitzsch et al., 2020; Ulitzsch, Yildirim‐Erbasli, et al., 2022). It is important to note, however, that it is not always possible to use response times for modelling careless responding. In this study, for example, only the response times for the entire questionnaires were available, and these were influenced by “other” options for some questions where participants could enter text. These questions were not important for the study at hand, but if text was entered, the times were automatically longer. In addition, technical problems during data collection led to some implausible response times.

In addition, one could use indices like the person‐fit indices discussed at the beginning of this paper to further validate the careless state. While this is not essential for the approach presented in this paper – where the states are predefined as careless or attentive using model constraints – it could still enhance classification in step 3 if there was significant uncertainty in the step 1 classification. However, using these indices may be especially interesting for validating the careless states in the more exploratory approaches mentioned above, where the careless state is not predefined but is interpreted as such after examining the states.

Note that the presented approach focuses on identifying and investigating C/IER occurrence in EMA data. Another important issue yet to be addressed is how to handle identified C/IER observations when addressing substantive research questions with EMA data. For now, we recommend conducting sensitivity analyses that evaluate the robustness of conclusions against adjustment for presumed C/IER. For C/IER adjustment, identified observations can be removed or down‐weighted according to their posterior C/IER state probabilities (; Ulitzsch, Shin, & Lüdtke, 2023). It is important to note, however, that doing so rests on the strong assumption that the occurrence of C/IER is not related to the construct to be measured. This assumption is, for instance, violated when researchers are interested in studying fluctuations in mood and C/IER is more likely to occur when respondents are in a negative mood. Then, filtering out identified C/IER observations may induce non‐ignorable missing values and, thus, potentially bias parameter estimates (Ulitzsch, Pohl, et al., 2023). While approaches exist that overcome this limitation by accounting for respondents' tendency to exhibit C/IER in the estimation of parameters of interest (Deribo et al., 2021; Ulitzsch et al., 2020; Ulitzsch, Pohl, et al., 2022), their integration with standard EMA analysis methods is, to date, unexplored.

In addition, future research could investigate how effectively C/IER can be differentiated from respondents' interactions with the scales that mimic but are not identical to C/IER (e.g., when some few respondents use the scale in a highly idiosyncratic way). Currently, researchers can already inspect individual transition plots, and if a person is consistently in the C/IER state, they could inspect the respective response patterns and think twice before labelling the person as careless. However, it is crucial to note that, in any case, for such instances, measurement invariance is violated and needs to be addressed (see, e.g., Vogelsmeier, Vermunt, van Roekel, & De Roover, 2019).

One limitation of the application is that we included participant‐level predictors for the transition probabilities in the LMM, which was only possible by repeating the participant‐level scores at every measurement occasion. This approach is not optimal – especially for analyses that extend beyond the exploratory purposes of the present study – because the sample size is artificially inflated. Thus, the accuracy of the findings is affected. This could be seen for the predictor *CR_check*, which was significantly related to transition probabilities but was practically uninformative regarding the likelihood of attentive responding and C/IER. A better approach would be to use a mixture latent Markov model (Crayen et al., 2017; Vermunt, 2008) with participant‐level predictors in step 3. Mixture LMMs cluster participants based on the most prominent differences in the transition patterns (e.g., participants who frequently transition between states versus those who are always in the C/IER or attentive state respectively). Participant‐level predictors can subsequently be included to predict participant‐specific cluster membership. However, to the best of our knowledge, it is currently not possible to estimate mixtureLMMs while also accounting for unequal intervals using continuous‐time latent Markov modelling in open‐source software, and for this article we aspired to present an open‐source estimation of the proposed methodology. However, if desired, step 3, including the additional mixture component, could be performed in Latent GOLD (Vermunt & Magidson, 2021). Example applications of mixture latent Markov modelling in step 3, including syntax, can be found in Vogelsmeier, Vermunt, Keijsers, and De Roover (2021) and Vogelsmeier, Vermunt, and De Roover (2023).

## AUTHOR CONTRIBUTIONS

**Leonie V.D.E. Vogelsmeier:** conceptualization; data curation; formal analysis; methodology; software; visualization; writing – original draft; project administration; supervision. **Irina Uglanova:** methodology; software; writing – review and editing. **Manuel T. Rein:** writing – review and editing; resources. **Esther Ulitzsch:** conceptualization; data curation; formal analysis; methodology; project administration; software; supervision; visualization; writing – original draft.

## FUNDING INFORMATION

This work was partially supported by the Research Council of Norway through its Centres of Excellence scheme, project number 33160.

## Data Availability

The data that support the findings of this study are openly available in OSF at https://osf.io/uj6sr/.

## Footnotes

1 In this study, we use the abbreviation C/IER and the two words careless and inattentive interchangeably.

2 Of course, respondents may also opt not to respond. Non‐response is another form of non‐compliance (see Jones et al., 2019; Morren et al., 2009; Ono et al., 2019; Wrzus & Neubauer, 2023). Whether individuals engage in non‐response or C/IER may depend on specific factors, such as the desire to achieve a high completion rate of questionnaires for a higher monetary reward. In this article, we focus exclusively on C/IER and not on non‐response.

3 It is important to note that the use of negatively worded items is not inherently beneficial. Some researchers have raised concerns that the inclusion of negatively worded items could affect psychometric properties, such as introducing measurement error. Additionally, there is debate about whether positively and negatively worded items truly assess the same construct (for an overview of critiques, see Dalal & Carter, 2014). Therefore, if not necessary because a single construct is measured, researchers should carefully think about the inclusion of negatively worded items. Nevertheless, as shown in the simulation study that follows, the employed confirmatory mixture model is also capable of detecting C/IER instances for different types of inattentive response patterns when all items from a unidimensional scale are worded in the same direction.

4 Note that the planned missingness design led to administering only positively worded items for 11% of the observations. Possible implications for the application results are described in the Discussion section.

5 Note that the data contained additional measures. These are, however, not relevant to this application and thus not further described. The interested reader is referred to the publicly available codebook: https://osf.io/ab579.

6 As explained in Vogelsmeier, Vermunt, and De Roover (2023), different selection procedures can lead to different results. Therefore, we performed a sensitivity analysis with forward selection; that is, we started with one predictor at a time and stopped as soon as the added predictors were no longer significant. The results were the same for forward and backward selection. In what follows, we report results obtained with backward selection only, but we provide code for both procedures in the analysis script.

7 Note that researchers are free to choose other intervals in their applications. For instance, when dealing with a sampling design with less frequent measurement occasions, larger intervals would make sense.

8 Note that interpreting transition probabilities for a specific interval and comparing them for different predictor values is the approach recommended by Vogelsmeier, Vermunt, and De Roover (2023). because the estimates of the CT‐LMM parameters – that is, the (effects on) transition intensities – are hardly interpretable.

9 It is important to note that including participant‐level predictors for the transition probabilities in the LMM is only possible by repeating the participant‐level scores at each of the participants' measurement occasions. The limitations of this approach are detailed in the Discussion section.

10 Recall that in the simulation study, we were interested in the quality of parameter estimates in step 1. For this, autocorrelation between factor scores or the Markov structure are irrelevant (Vogelsmeier, Vermunt, Bülow, & De Roover, 2021).

11 It is well understood that confirmatory mixture models provide more accurate parameter estimates when classes are more balanced (i.e., with an increasing C/IER rate) as well as with an increasing sample size. Therefore, we decided to keep these factors constant to keep the simulation design tractable and refer interested readers to previously obtained results provided in, e.g., Ulitzsch et al. (2020).

12 Nevertheless, note that model‐implied category probabilities and, hence, expected response patterns from attentive respondents with a middle location on the latent trait still exhibited subtle differences from the simulated C/IER respondents with a strong preference for the midpoint even under conditions without negatively worded items. Otherwise, separation of these two types of behaviours would be impossible. For instance, for the item life_ideal, the probability of selecting the midpoint category peaked around θ=‐0.30 with 0.53, and the probabilities for the two adjacent categories for this trait level were 0.21. For the same trait level, the probability of selecting the midpoint category on the item important_things was 0.38, and the probabilities for the two adjacent categories for this trait level were 0.18 and 0.34 respectively.

13 Having loadings decrease according to an exponential decay process is a somewhat arbitrary choice. Note, however, that because the order of observations is not taken into account in step 1, the specific form of changes in loading patterns will not impact results. What matters is the proportion of observations with specific loading patterns.

## DATA‐GENERATING ITEM PARAMETER VALUES

**TABLE A1:** Data‐generating item parameter values for respondents with category preferences in C/IER state.

α	$\kappa_{1}$	$\kappa_{2}$	$\kappa_{3}$	$\kappa_{4}$	$\kappa_{5}$	$\kappa_{6}$
1	3.86	1.36	0.64	0.24	−0.59	−2.72

*Note*: The parameter values were equal to the estimates of the application reported in the Empirical Application section.

**TABLE A2:** Data‐generating item parameter values for the attentive State.

	α	$\kappa_{1}$	$\kappa_{2}$	$\kappa_{3}$	$\kappa_{4}$	$\kappa_{5}$	$\kappa_{6}$
consider_happy	3.79	9.45	6.16	3.96	2.10	−0.93	−6.98
satisfied_life	4.10	10.20	6.46	4.20	1.84	−1.32	−7.34
change_many	−3.10	5.82	0.61	−1.02	−2.14	−4.31	−7.74
life_ideal	4.45	9.97	4.97	2.49	0.12	−2.97	−8.72
makes_happy	3.50	8.88	5.69	3.39	1.57	−0.95	−6.35
satisfied_situation	4.45	10.85	6.60	3.99	2.44	−0.74	−8.08
change_nothing	2.55	6.23	3.14	1.34	0.16	−1.48	−5.58
leaves_desired	−2.36	4.97	1.04	−0.26	−1.52	−3.19	−6.36
important_things	2.86	7.52	4.11	2.12	0.46	−1.80	−6.15
line_life	4.07	9.25	4.83	2.63	0.42	−2.55	−8.33

*Note*: The parameter values were equal to the estimates of the application reported in the Empirical Application section.
